# Identification of Microchip Implantation Events for Dogs and Cats in the VetCompass Australia Database

**DOI:** 10.3390/ani9070423

**Published:** 2019-07-05

**Authors:** Paul McGreevy, Sophie Masters, Leonie Richards, Ricardo J. Soares Magalhaes, Anne Peaston, Martin Combs, Peter J. Irwin, Janice Lloyd, Catriona Croton, Claire Wylie, Bethany Wilson

**Affiliations:** 1 Sydney School of Veterinary Science, Faculty of Science, University of Sydney, Sydney, NSW 2006, Australia; 2 Faculty of Veterinary and Agricultural Sciences, University of Melbourne, Werribee, VIC 3030, Australia; 3 School of Veterinary Science, University of Queensland, Gatton, QLD 4343, Australia; 4 Child Health Research Centre, University of Queensland, South Brisbane, QLD 4101, Australia; 5 School of Animal and Veterinary Sciences, University of Adelaide, Roseworthy, SA 5371, Australia; 6 School of Animal and Veterinary Science, Charles Sturt University, Sutherland Laboratories, Wagga Wagga, NSW 2650, Australia; 7 School of Veterinary and Life Sciences, Murdoch University, Murdoch, WA 6150, Australia; 8 College of Public Health, Medical and Veterinary Science, James Cook University, Townsville, QLD 4811, Australia

**Keywords:** cats, dogs, microchip, strays, VetCompass Australia

## Abstract

**Simple Summary:**

The implantation of a microchip can maximise an animal’s chance of being returned to its owners, if separated, but is also a statutory requirement for companion animal owners in many jurisdictions across Australia. This study of the electronic patient records of 1000 randomly selected dogs and cats in the VetCompass Australia database revealed that the median age at microchip implantation was 74.4 days for individual dogs and 127.0 days for individual cats. Further exploration into the reasons for later microchipping in cats may be useful in aligning common practice with legislative requirements.

**Abstract:**

In Australia, compulsory microchipping legislation requires that animals are microchipped before sale or prior to 3 months in the Australian Capital Territory, New South Wales, Queensland and Victoria, and by 6 months in Western Australia and Tasmania. Describing the implementation of microchipping in animals allows the data guardians to identify individual animals presenting to differing veterinary practices over their lifetimes, and to evaluate compliance with legislation. VetCompass Australia (VCA) collates electronic patient records from primary care veterinary practices into a database for epidemiological studies. VCA is the largest companion animal clinical data repository of its kind in Australia, and is therefore the ideal resource to analyse microchip data as a permanent unique identifier of an animal. The current study examined the free-text ‘examination record’ field in the electronic patient records of 1000 randomly selected dogs and cats in the VCA database. This field may allow identification of the date of microchip implantation, enabling comparison with other date fields in the database, such as date of birth. The study revealed that the median age at implantation for dogs presented as individual patients, rather than among litters, was 74.4 days, significantly lower than for cats (127.0 days, *p* = 0.003). Further exploration into reasons for later microchipping in cats may be useful in aligning common practice with legislative requirements.

## 1. Introduction

Microchipping of companion animals is an important part of the role of Australian veterinarians. A microchip is a small radio-frequency identification device (RFID) enclosed in an inert capsule and implanted into the subcutaneous tissue of an animal (usually a dog, cat or horse) to provide permanent electronic identification of the animal. Following implantation, microchips can be read by specialised scanners with a very high degree of accuracy [1,2]. Through the use of specialised databases, the number read from the microchip can be linked to owner details and to animal health records, such as vaccination and neuter status.

A primary goal of permanent identification of animals is to allow owned stray individuals to be re-united with their owners. The current annual number of dogs admitted to Australian municipal council pounds, animal welfare organization shelters and animal rescue groups has been estimated to be in excess of 200,000 [3]. While this number also includes canine surrenders, the high reclamation rate in that study (48%) suggests that at least half of these dogs were not intentionally separated from their owners. With respect to cats, one study showed nearly 7000 cats admitted over a year to the Royal Society for the Protection of Cruelty to Animals (RSPCA) shelters as uninjured or injured strays in one Australian state (Queensland) [4]. The proportion of microchipped cats reclaimed by owners was 33–61%, but was only 5% for unchipped cats. Data from both Australia [4] and overseas [5] demonstrate that microchipped stray animals have a greater chance than non-microchipped stray animals of being reunited with their owners. As the electronic patient records (EPRs) include information about microchipping, the VetCompass Australia (VCA) database is a useful tool for exploring common practice for veterinary microchip implantation.

Microchipping is a statutory requirement for companion animal owners in most states in Australia. According to the RSPCA [6], compulsory microchipping legislation requires an animal to be microchipped before sale or prior to 3 months of age in the Australian Capital Territory, New South Wales, Queensland, South Australia and Victoria, and by 6 months of age in Western Australia and Tasmania.

The advent and wide use of electronic veterinary patient records has facilitated post-hoc epidemiology investigations, such as those emerging from the VetCompass Animal Surveillance project. First created by the Royal Veterinary College, UK in collaboration with the University of Sydney, this project collates electronic patient records, including individual animal microchip numbers, from 1100 primary care veterinary practices in the UK, to maintain a database for veterinary and animal welfare related epidemiological studies [7]. The similarly modelled VCA is the first Australia-wide surveillance system that collates anonymized EPRs from Australian primary care and specialist veterinary practices [8].

Dogs and cats may attend more than one veterinary clinic throughout their lifetimes, and the use of a microchip number as a permanent identifier allows linkage of the health records from multiple clinics. Especially important when estimating the prevalence of chronic diseases, this ensures double counting does not occur and permits the linkage of early life records to late life records. Therefore, to optimise the use of the data available in the VCA database, it is important to describe the implementation of microchipping in companion animals. To develop a methodology for identifying microchip implantation events in the free-text ‘examination record’ field of the database is a priority. This field may identify a microchip implantation date, enabling comparison with other date fields in the database, such as date of birth, and other significant health events such as neutering surgeries and vaccinations, as recorded in the examination record field. Our methodology helps to enable optimal use of the data available in the VCA database. The primary purpose of the paper is to be a research note targeted at those using the VCA database and the growing number of similar veterinary databases that draw data from electronic patient records.

## 2. Materials and Methods

### 2.1. Data Management

Dogs and cats with a recorded veterinary consultation in the year 2017 were selected as a cohort. These represented 2,222,859 rows in the database, indicating a potential maximum of 2,222,859 veterinary consultation events. However, a single visit to a veterinarian often results in the generation of multiple rows. After exclusion of data from rows without a 15 character numeric entry in the microchip field (the length and format of a valid microchip for Australia), 1,631,322 rows remained, associated with 172,443 unique patient IDs (132,924 (77.1%) dogs and 39,519 (22.9%) cats). However, there were only 171,207 unique microchip fields (131,972 dogs and 39,235 cats). This suggests that the same animal presented at multiple veterinary clinics and was issued a new patient ID at each one. 

To identify the most successful search strategy (Search A) for identifying records containing the description of, billing of, or some other indication of a microchipping event performed on a particular date, the EPRs for the 172,443 patients with a valid microchip number were searched using the following simple terms: ‘implant,’ ‘mchip,’ ‘m chip,’ ‘microc,’ ‘m/c,’ ‘micro-c’ and ‘micro c.’ Records containing these strings of letters would also be identified, e.g., ‘microc’ would identify ‘microchip.’ Search A was defined as the single simple term search that returned the most records.

### 2.2. Sample Size Calculation

To determine the percentage of microchip procedure dates detected by Search A in animals with a microchip, 1000 records from the 172,443 results were selected randomly, using the RAND() function in MS Excel, which operates on the Mersenne Twister algorithm. These were then coded manually as either representing or not representing a microchip implantation event. Common reasons for a record being returned despite not describing a microchip implantation were also identified (see Table 1). To investigate the utility of Search A for identifying microchip implantation events in animals lacking a recorded microchip number, a separate sample of 1000 EPRs selected by random number generator from records with invalid, incomplete or blank microchip numbers (henceforth simply referred to as “invalid”) was searched using Search A, and coded manually as above.

Using the sample size calculator at http://epitools.ausvet.com.au it was estimated that 208 cats would be needed to compare the valid to the invalid microchip groups, to detect a difference between 9% microchipped (as estimated from Reference [4]) and 19% (a 10% increase in the other group) with a power of 80% and a confidence level of 0.95. This corresponded roughly to the 229 cats that would be expected in a 1000 animal sample based on the overall proportion of dogs and cats in the data. 

The probability that the animal record that returned a search hit for microchip utilising search A (true positives + false positives) did actually have a microchip implanted on that day (true positives) was calculated as the fraction of returned records that included an implantation event [9]. Reasons for false positives (rows flagged by the search term that did not contain mention of a microchip implantation event in the ‘examination text’ field) in the records with valid microchip numbers recorded and invalid records were compared using tests for the significant difference of proportions, using a chi-squared test with R statistical software.

The microchipping events were then classified by species (dog or cat), and whether microchipping was performed on an individual animal (an animal with its own patient ID) or on a litter (the animal was recorded in the dam’s EPR or as part of a litter issued a group patient ID). For an individual animal, the age at implantation was determined by subtracting the date of birth from the date of the implantation event. The ages at which dogs and cats were microchipped were compared using a two sample *t*-test with a logarithmic transformation to attain normality of the age distributions.

Finally, microchipping events were classified according to whether other significant veterinary interactions were occurring on the implantation date. Tests for the significant difference of proportions (chi-squared test) were used to evaluate differences in the proportion of cats and dogs who were microchipped at the same time as other significant veterinary interactions.

## 3. Results

The success of each search term in identifying potential implantation events is recorded in Table 2.

The search term ‘microc’ identified the most records. The value of additional terms was considered by evaluating whether they identified overlapping sets of patients. 

The key terms we pre-specified were identified in the database to different extents (ranging from comprising 0.05% to 86.3% of the hits). The variant ‘microc,’ which would identify the full word ‘microchip’ and variants, detected by far the most records, and these EPRs were selected for further investigation. The characterisation of the randomly selected EPRs is shown in Table 3 below, and is compared with a sample of 1000 records from the cohort with an invalid entry in the microchip field.

The review of ‘microc’ identified that 25.7% of hits in microchipped animals represented an animal receiving a new microchip implantation and 74.3% did not. Extrapolating from our random sample to the full 43,365 records (see Table 2) returned from the ‘microc’ search, we might expect this search to reveal approximately 11,000 implantation events among our 172,443 unique patient IDs. The positive predictive value (PPV) of the search results revealing a microchip implantation event in microchipped animals was 0.257. However, improving the identification of these events would require significant refinement of the methods.

### 3.1. Comparison to Records with Invalid Microchip Fields

Records with invalid microchip fields were included in the results for the ‘microc’ search term that did not represent an implantation event. Among invalid records, the PPV of a ‘microc’ search result (true positives + false positives) representing an implantation event (true positives) was 0.155. This was significantly lower than 0.257, the positive predictive value found for the valid, 15 digit microchip records (χ^2^ = 31.184, *p* < 0.001). 

Non-implantation events were also classified (See Table 3), and comparison of the reasons for false positive search results for the valid and invalid microchip field cohorts is presented in Table 1. Significantly fewer search results from the invalid records sample represented a non-completed auto-generated form including the word ‘microchip’ (χ^2^ = 10.63, *p* = 0.001), or a reference to a pre-existing microchip (χ^2^ = 4.819, *p* = 0.028), than did the sample with completed microchip fields. Significantly more search results from the invalid records sample represented a plan for future microchip implantation (χ^2^ = 23.702, *p* < 0.001), a successful identification of a stray animal (χ^2^ = 32.271, *p* < 0.001), an unsuccessful identification of a stray animal (χ^2^ = 25.38, *p* < 0.001), an animal reported lost (χ^2^ = 9.141, *p* = 0.002), a noted absence of a microchip (χ^2^ =74.754, *p* < 0.001) and an owner refusing microchipping (χ^2^ = 17.216, *p* < 0.001) than in the records with valid microchip fields. Other reasons occurred too infrequently to be evaluated statistically or were not significant.

Among the 257 implantation events associated with a valid microchip field, 11 were classified as litter microchipping events, whereas, among invalid microchip fields, 36 (of 155) were classified as litter microchipping events (χ^2^ = 32.49, *p* < 0.001).

### 3.2. Timing of Microchip Implantation Events

The concurrence of the 412 microchip implantation events (i.e., combined number of valid and invalid microchip numbers) with other interactions (or planned interactions) that veterinary staff recorded in that EPR is shown in Table 4.

Records showed a valid date of birth field, and a subsequent valid visit date, allowing for a calculation of the age of the animal at the implantation event for 254/257 (98.8%) implantation events with the valid microchip fields, and 137 for the 155 (88.4%) implantation events in invalid records.

**Figure 1:**
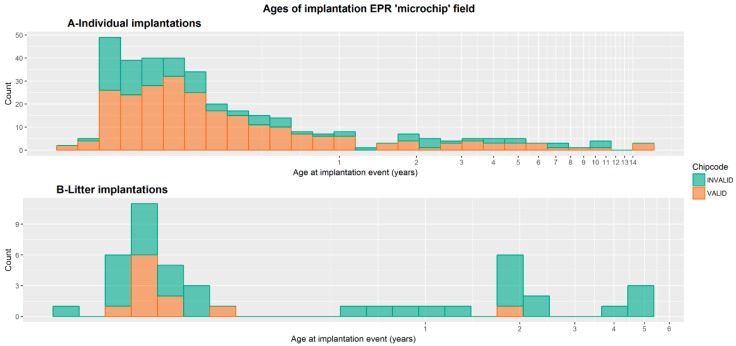
A and B: Showing the apparent age at microchip implantation for animals with both valid and invalid microchip fields in the electronic patient records (EPRs) for (**A**) individuals and (**B**) litters. Orange portion of bars = electronic patient records with a valid microchip field, green portion of bars = EPRs with an invalid microchip field. Age for litter vaccination would be the dam’s age if the implantation event was recorded within her EPR.

The median age at implantation for individual events was 89.7 days (IQR = 134.97 days). Litter vaccinations are all likely to occur early, prior to the sale of puppies to individual owners, and so the higher ages seen in Figure 1B are referring to the age of the dam.

**Figure 2:**
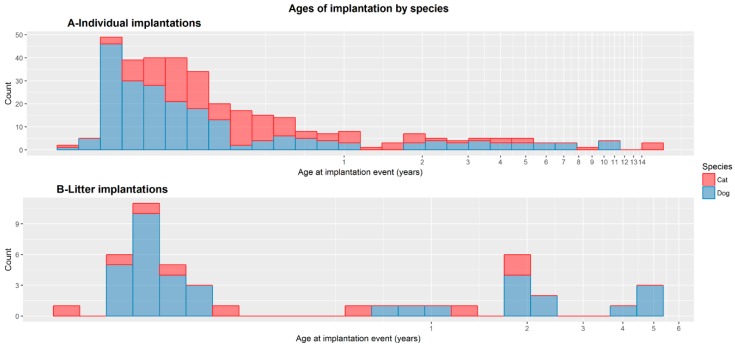
A and B: Showing the apparent age at microchip implantation for both dogs and cats in the electronic patient records for (**A**) individuals and (**B**) litters. Red = cats, blue = dogs.

The median age at implantation for individual dogs was 74.1 days (IQR = 95.23 days), and for cats was 127.0 days (IQR = 131.45 days) (See Figure 2). A two sample *t*-test of logarithmic transformed ages showed this difference is statistically significant (*t* = 2.9009, *p* = 0.003). Analysis of the proportions of concurrent events in Table 4 showed that the proportion of cats microchipped at the time of a vaccination was significantly lower than the proportion of dogs microchipped at vaccination (χ^2^ = 55.376 *p* < 0.001) but the proportion of cats microchipped at the time of spay (χ^2^ = 17.519, *p* < 0.001) or castration (χ^2^ = 32.45, *p* < 0.001) was significantly higher than dogs microchipped at the time of these surgeries.

### 3.3. Substitution of Search Term ‘microch’

Among the EPRs sampled with both valid and invalid microchip fields, the string detected by the search term ‘microc’ was text referring to a microchip in over 99% of cases. However, in nine cases, the search found a different word, such as ‘microcyte’, ‘microcytic’ or a misspelling of microscope. Given these false positives, the results of the search term ‘microch’ was compared for efficiency to those of the search ‘microc’ for the entirety of records with 15 digit microchips. This revealed that 126 records were identified by ‘microc’ but not ‘microch.’ The words accounting for these 126 records are shown in Table 5.

Given that around 12% of the records by the longer search do represent the word ‘microchip,’ it may be advantageous to include common false matches and misspellings using a Boolean ‘NOT’ term or similar, rather than narrow the search to ‘microch,’ which would not identify these 12% of records

### 3.4. Analysis for Microchip Numbers Duplicated in the EPR for Multiple Patients

Analysis revealed that there were 1189 valid microchip numbers associated with two different patient numbers in the VCA database. For most of these records (746, 62.7%) both patient numbers were associated with the same species, breed and sex of animal, and most likely represented patients seen at multiple clinics. A further 335 (28.2%) of these records showed the same species and sex, but differed by breed, some of which differed only slightly (such as a Labrador in one record and a Labrador cross in another). However, others may represent a difference in staff opinion (such as a domestic long hair vs. a domestic medium hair cat) or a difference in variant or terminology (such as Jack Russell terrier or Parson Russell terrier). Other examples include physically similar breeds (such as Siberian husky and Alaskan malamute), where different breed names might be applied to the same animal.

A further 39 (3.28%) differed by sex. Of these animals, 18 were cats, which may represent a point of confusion for owners or veterinary staff, or else incorrect data entry. It is possible that these records represent some intersex animals, although this was not explored. There were 34 records, 31 of which were dogs that varied by both breed and sex. These discrepancies may represent simultaneous occurrences of both types of error discussed previously, although cases such as male German shepherd dogs paired with a female Jack Russell terrier cross and a male Labrador retriever paired with a female Shih Tzu cross might be more likely to represent a typographical error in the microchip field. A remaining 35 records matched dogs with cats and presumably represent microchip field entry errors, or an incorrect species entry at one clinic.

There were 18 further microchip numbers that matched with three different patient IDs. In 15 of these cases, all three records matched for species and sex, and matched or nearly matched for breed. There were also two numbers that matched five IDs.

## 4. Discussion

This study aimed to identify microchipping events within EPRs of the VCA database, and investigate when microchip implantation was occurring relative to legislative requirements. After evaluating several search terms, the term ‘microc’ was selected, and 1000 EPRs containing this search term were manually reviewed. A large number of these EPRs appear to contain the term because automated forms generated the word ‘microchip’ in the record, such as when a pathology report or referral letter was transcribed into the EPR. It may be possible to code searches to avoid picking up these records in the future.

There were multiple other reasons for the term ‘microc’ appearing in the EPRs. This finding confirms that the interactions between veterinary staff, pet owners and microchips go beyond patient microchip implantation, including assisting clients with administrative tasks such as change of details and changes of ownership, and scanning animals owned by new clients to confirm the implantation of a microchip. This also included identifying or attempting to identify stray and injured animals presented to veterinary clinics by accessing the clinic’s records or large, centralized microchip data services. This last task may be of particular significance for practice management, as it presents a second opportunity for the microchip number to be linked to current contact information for the owner in cases where owners may have moved or changed address since the microchip was implanted. One recent Australian study identified that over a third of the stray animals presented to shelters had problems with their microchip data, such as being registered to a previous owner or organisation (47%), having had all phone numbers either incorrect or disconnected (29%), or the microchip not being registered (14%) [4]; this second chance could prove life-saving in many cases. 

Among stray animals presented to veterinary clinics in the current samples, two out of two of the patient IDs with valid microchip fields and 39 out of 66 of the patient IDs with invalid microchip fields were classed as successfully identified. This suggests a rate of microchipping considerably higher than that found by Lancaster et al. [4], which found that only 28% of dogs and 9% of cats entering a shelter were microchipped. However, it is important to acknowledge potential limitations of the accuracy of the estimate in the current report. Firstly, only those EPRs for stray animals which included the search term ‘microc’ were eligible for random selection. This might also explain the relatively low absolute number of stray animals in the sample of those with a valid microchip field. If a stray is immediately identified by staff as a patient of the clinic, whether it was scanned for a microchip may not be recorded. Secondly, the cohort of stray animals presented to VCA clinics and those presented to shelters or councils might be expected to differ. Certainly, the current EPRs did sometimes note that a stray dog that the clinic could not identify had been passed on to a council ranger, whereas stray dogs that were identified through microchip data as patients of the clinic were often picked up directly from the clinic. Thirdly, the current study did not require evidence of current contact information of the owner for successful identification to have occurred, as a previous owner may yet be able to contact or provide contact details for the current owner. Finally, the VCA database includes data on rescued animals that have undergone mandatory microchipping before leaving shelters, but we have no means of identifying them specifically.

The study identified 263 microchip implantation events for dogs (or litters of pups) and 149 implantation events for cats (or litters of kittens). As the RSPCA Australia estimates that there are 4.8 million companion dogs in Australia and 3.9 million owned cats in Australia (ratio dogs:cats = 1.2) [10], the ratio of 1.8 dog implantation events for every one cat might suggest a lower implantation rate in cats. At the same time, the underrepresentation of cats is consistent with both Australian [4] and international [5] literature, which notes higher microchipping rates and higher rates of recommendation [11] in dogs. This imbalance may, in part at least, result from the relative delay in microchipping of cats.

Our data showed that the median age at implantation for individual events for dogs is 74.4 days, and is significantly lower than that for cats (127 days). Thus, half of dogs are microchipped just within the 12 week (3 month) legislative deadline in most Australian states and territories, but more than half of cats remain unchipped by the legislated deadline. As noted above, it seems most common for puppies to be microchipped at the time of a vaccination, but for cats to be microchipped at the time of neutering. This may be due to kittens taking longer on average to reach a desired minimum size for microchipping, less urgency in clients’ demand for microchipping cats, clients acquiring kittens at an average older age, or acquiring kittens from a source less likely to have microchipped them as part of a litter event. This finding may even reflect veterinarians’ preferences, such as their having less confidence restraining cats for microchipping without general anaesthesia, or increased perception of cats requiring the pain relief provided by general anaesthesia. Further exploration into reasons why cat microchipping is performed later than in dogs may be useful in bringing common practice in line with best practice.

Another benefit of studying the microchipping data in the VCA database was to explore the question of how many animals are presented at more than one veterinary clinic, and therefore have more than one patient ID associated with their microchip over the course of a year. While it was not always possible to distinguish multiple clinic visits from mistyped microchip records, our best estimate is that of the 171,207 unique microchip fields (131,972 dogs and 39,235 cats), at least 1000 were associated with patients seen at multiple clinics. We recommend that, depending on their particular study design, those using VCA for epidemiological research check their cohorts for duplicated microchip numbers and consider how best to minimise potential bias.

Overall, this study has shown that, while identifying microchipping events manually in the VCA database is possible, it is labour intensive due to low positive predictive values of the trialled searches, requiring extensive manual examination of records. Searching for the sale of microchip products was not explored, and, while such searches are likely to have a high PPV for microchip implantation events, it may not have a high negative predictive value, as the brand of microchip may not always be noted. Nevertheless, given that stray animals are a problem in Australia, and that reunification with their owners is the best outcome for most stray animals, further research into and monitoring of microchipping practices within the veterinary population of dogs and cats is merited. Additionally, this study revealed activities by veterinary clinics to identify and reunite stray animals with their owners. From an animal welfare perspective, this practice is to be encouraged, as this gives these strays an opportunity to be linked to current owner contact details.

## 5. Conclusions

This study of the electronic patient records of a 1000 randomly selected dogs and cats in the VetCompass Australia database revealed the apparent median age at implantation for individual events for dogs is 74.4 days, and is significantly lower than that of cats (127.0 days). Further exploration into the reasons behind microchipping being performed later in cats may be useful in bringing common practice in line with legislative requirements.

## Figures and Tables

**Table 1:** Comparison of reasons for a positive search result that did not represent a microchip implantation event between Valid Microchip Field cohort and Invalid Microchip field cohort.

Reason Classification	Valid Microchip Field	Invalid Microchip Field	Chi Sq	*p*-Value
Auto-generated form of clinical parameters to be populated including the ‘microchip’ if present	61.64%	45.62%	10.63	0.001
Confirmation or notation of pre-existing microchip within the free-text of an EPR	27.32%	19.43%	4.819	0.028
A plan or scheduling of microchipping in the future	3.36%	8.65%	23.702	<0.001
Identification of a stray/injured animal				
*Successful*	0.27%	4.62%	32.271	<0.001
*Attempted*	0.00%	3.20%	25.38	<0.001
Animal reported lost (presence or absence of chip noted)	0.00%	1.30%	9.141	0.002
Absence noted/microchip not found	0.67%	10.31%	74.754	<0.001
Owner refusal to have animal microchipped noted without evidence of future plan	0.00%	2.25%	17.216	<0.001

**Table 2:** The number of records (potential microchip implantation events) returned from a cohort of 1,631,322 VetCompass Australia electronic patient records associated with a veterinary consultation in the year 2017 and a 15 digit number recorded in the ‘microchip’ field.

Search Term	Number of Records	Number of Patient Unique Identification Numbers	Number of Patient IDs also Found by Searching for ‘microc’
‘implant’	2009	1710	1246
‘mchip’	554	521	315
‘m_chip’	31	31	17
‘microc’	43,365	26,145	-
‘m/c’	2019	1832	1171
‘micro-c’	15	15	11
‘micro_c’	28	28	19

**Table 3:** Classification of 1000 VetCompass Australia (VCA) electronic patient records returned by the search term ‘microc,’ both for records with valid 15 digit microchip number recorded in the ‘microchip’ field and records with an invalid entry, and common causes of identifying records (other than the record describing an implantation event).

Reason for Detection of Individual Records Using ‘microc’ Search Term	Valid Microchip Field	Invalid Microchip Field
Microchip implantation event	257	155
A blank pre-populated clinical parameter form with space for ‘microchip’ to be entered	458	385
Confirmation or notation of pre-existing microchip within the free-text of an electronic patient record (EPR)	203	164
A plan or scheduling of microchipping in the future	25	73
Assisting clients with administrative tasks, e.g., change of ownership	9	7
A notation requesting microchip number be collected for clinic’s internal records which was not completed	8	9
Identification of a stray/injured animal		
*Successful*	2	39
*Attempted*	0	27
Travel requirements, e.g., vaccination certificate	4	2
Technical problem with a microchip	1	1
Attempt to contact previous owners/veterinarians/veterinary history	2	1
Complication of microchipping implantation	6	0
Other words/typographical errors	2	7
Animal reported lost (presence or absence of chip noted)	0	11
Absence noted/microchip not found	5	87
Owner refusal to have animal microchipped noted without evidence of future plan	0	19
None of the above	18	12

**Table 4:** Veterinary interactions in electronic patient records concurrent with 365 individual animal microchip implantations in 1000 randomly selected results to the search term ‘microc’ with a valid microchip number (implantations = 246) and an invalid microchip number (implantations = 119).

Concurrent Events	Valid	Percent of Valid	Invalid	Percent of Invalid	Total	Dogs	Percent of Dogs	Cats	Percent of Cats
vaccination	152	61.79%	84	70.59%	**236**	179	79.20%	57	41.00%
spay	29	11.79%	15	12.61%	**44**	14	6.19%	30	21.60%
castration	46	18.70%	10	8.40%	**56**	15	6.64%	41	29.50%
other surgery	5	2.03%	0	0.00%	**5**	3	1.33%	2	1.40%
other consult	5	2.03%	3	2.52%	**8**	6	2.65%	2	1.40%
none	9	3.66%	7	5.88%	**16**	9	3.98%	7	5.00%

**Table 5:** Words that were found by searching the 2017 electronic patient records of the VetCompass Australia database using ‘microc’, but not when using ‘microch’.

Words or Intended Words Found by ‘microc’ but not ‘microch’	
Microcardia	8
Microcautery	2
Microchip *	14
Microcilia	2
Microclimate	1
Microclone	3
Microclot	2
Microconidia	2
Microconvex	32
Microcrystals	1
Microcu	2
Microcysts	1
Microcytes	4
Microcytic	17
Microcytosis	14
Microlax	5
Microscop *	15

* There were various misspellings but these were the words or root of words intended.
